# The Effects of Intranasal Oxytocin Administration on Sensitive Caregiving in Mothers with Postnatal Depression

**DOI:** 10.1007/s10578-016-0642-7

**Published:** 2016-04-21

**Authors:** Beth L. Mah, Marinus H. Van Ijzendoorn, Dorothee Out, Roger Smith, Marian J. Bakermans-Kranenburg

**Affiliations:** 1 Mothers and Babies Research Centre, Hunter Medical Research Institute, Lookout Road, New Lambton Heights, NSW 2305 Australia; 2 Centre for Child and Family Studies, Leiden University, PO Box 9555, 2300 RB Leiden, The Netherlands

**Keywords:** Oxytocin, Depression, Postpartum, Postnatal, Maternal behaviour

## Abstract

Postnatal depression (PND) is common and negatively affects the mother–infant relationship; oxytocin (OT) has been found to have positive effects on parenting, although psychiatric disorders may reduce these effects. Thus, we explored the role of OT in mothers diagnosed with PND. A within-subject, randomized controlled double-blind design was used to test the effects of nasal administration of OT or placebo on sensitive caregiving. The outcome measures were perceptual and caregiving responses to prerecorded cry sounds, as well as observed maternal sensitivity. We found that in the OT condition mothers with PND were more likely to rate an infant cry as more urgent and they were more likely to indicate they would chose a harsh caregiving strategy in response. There was no effect of OT on maternal sensitive interaction with their own baby. Further research is required prior to consideration of OT administration in depressed mothers of infants.

## Introduction

Infants signal their needs to elicit adequate caregiving in numerous ways, and the percentage of time that a caregiver is attuned to the infant’s signals has been found to be a powerful predictor of infant socio-emotional development [1]. Infants are able to use a varying repertoire of cues to achieve caregiving, changing their tactic if one strategy fails to achieve the desired result [1]. Ainsworth contributed much to our knowledge in this area, recognizing the importance that for a sensitive response to infant signals caregivers need to accurately perceive and interpret an infant’s signal, and respond appropriately to it [2]. Together, these capacities have been labeled Sensitivity. Mothers vary in their caregiving sensitivity, and those who display high maternal sensitivity are more likely to have an infant with a secure attachment and better academic outcomes [3–6]. The current study explores the role of OT in the provision of sensitive caregiving by mothers diagnosed with PND, using a within-subject, randomized controlled double-blind design with nasal administration of OT or placebo.

Sometimes an infant cue is not enough to provoke a needed response. One common situation where this occurs is when a mother has a diagnosis of PND. Arguably two of the most important sensory modalities a caregiver uses to perceive the needs of an infant are the visual perception of the infant’s facial expression and the auditory perception of infant vocalizations including crying. Depressed mothers are less likely to accurately identify infants’ happy facial expressions [7] and they have a decreased neural response to positive infant signals, such as smiling [8], compared with non-depressed controls. Various features of a newborn’s cry communicate to the caregiver the state of the baby, and the urgency of its need for care. Infants born with various medical complications signal their distress with a higher pitched cry compared with well newborns [9, 10]. Observation of caregiver response indicates that in general greater soothing efforts are elicited by higher pitched infant cries [11], however, depressed mothers are less likely to perceive the saliency of higher pitched infants cries [12].

In terms of maternal ability to interpret infant communications, depressed mothers have been found to generally perceive their infants as more difficult [13, 14], interpret the bond shared as more negative [15], have poorer perceptions of their infants’ adaptability [16] and have lower reflective capacity when considering the meaning of their infants’ communication [17]. Finally, PND affects the parental response to the infant negatively. At the most serious end of a spectrum of caregiving, depressed mothers are more likely to neglect or use aggressive parenting styles [18], and their infants’ mortality rates are higher [19] than those of non-depressed controls. Other important aspects of caregiving are also affected. Depressed mothers have been found to show less positive engagement [13], to have poorer interactions [20], and to be less responsive [14], less attuned [21], less sensitive, and more intrusive with their infants [22]. They also have infants with higher rates of attachment insecurity [22].

The relationship between plasma OT level and aspects of parenting have been studied in community populations. It has been found that plasma OT level during pregnancy and during the postnatal period is positively associated with sensitive parenting [23, 24]. Parental plasma OT level raises during contact with their infant generally but more marked increases occur for parents who show greater sensitivity of parenting [25]. It seems that other parental aspects contribute to differing levels of OT increase after contact with an infant. Mothers who have high levels of affectionate contact with their babies and fathers who are highly stimulating in their play have the greater OT rise after contact [26]. Mothers’ temperaments influence the OT rise; those sensitive to emotion and less schedule driven have greater plasma OT increases than mothers with different temperamental styles [27]. Finally, in terms of adult attachment classification, mothers classified as secure have the greatest plasma OT rise in response to contact with their baby than those whom are classified as non-secure [28].

The link between OT and depression remains unclear. Correlational studies have found lower serum levels of OT in depressed in-patients [29]. Conversely, higher OT levels have been found in adults with major depression [30, 31]. However, correlational research specific to mothers with depression is sparse. Lower plasma OT level during mid-pregnancy predicts a risk for the subsequent development of PND [32, 33]. Outcomes for children have also been measured when considering the link between OT and PND. By the age of six, children whose mothers were chronically depressed were four times more likely to be diagnosed with an axis I disorder. They also had lower empathy and social skills than control peers. Interestingly, when salivary OT levels were measured in both the children and their chronically depressed mother, levels were lower than controls [34].

Considering the literature exploring associations between OT administration and parenting behaviors, in a community sample, the administration of intranasal OT showed positive impact on parenting behaviors. Fathers displayed greater responsive structuring and were less hostile in the OT condition compared to the placebo condition when interacting with their young children [35]. In a study of non-parents, testing responses to a newborn cry sound, administration of intranasal OT resulted in lower amygdala activation and presumably lower anxiety whilst simultaneously increasing activation in empathy related regions [36]. In another community study of non-parents the effects of OT were explored in association with childhood experiences. Participants were asked to squeeze a hand-grip dynamometer to a previously learned mid-point, after hearing infant cry sounds. Intranasal OT decreased excessive use of handgrip force but only in those who had not experienced harsh discipline [37]. In psychiatric clinical samples there is a trend of diminished OT effectiveness for subjects diagnosed with various conditions associated with untoward childhood experiences [38]. Thus exploring the effects of OT administration on parenting in mothers with a diagnosis of PND is important.

In a previous report on the current sample administration of intranasal OT caused lower self-reported mood in depressed mothers but at the same time they rated the relationship with their infants as more positive compared with a placebo nasal spray [39]. Given the importance of sensitive parenting and concerns that PND decreases sensitive responsiveness, we tested the following two hypotheses: (1) We expect that the administration of intranasal OT enhances a depressed mother’s ability to perceive the urgency of cry sounds, and enhances her intention to choose a sensitive care giving strategy after presentation of cry stimuli; (2) we expect that intranasal OT administration results in improvements in maternal sensitivity in mothers with PND as observed in interaction with her own young infant. Given the previously documented moderating effect of childhood experiences [37, 38, 40], we explore whether the effects are stronger for mothers with non-abusive backgrounds compared to mothers with childhood experiences of physical abuse.

## Method

### Procedure

Twenty-five mothers (mean age 28.24 years, *SD* = 5.93, range 19–38) participated in the double-blind, placebo-controlled, within-subject design. All participants received intranasal OT and placebo on separate visits to investigate the effects of OT on response to infant crying and the interaction with their child. The participants were recruited from various health agencies, and all had a diagnosis of PND. Infants participating in the study were aged between 3 and 12 months (mean age 6.22 months, *SD* = 2.44). Stenlake Compounding Chemist (Bondi, Australia) produced both the OT and placebo, bottling the two in identical containers for double blind purposes. Roughly half the participants received OT during the first visit. Randomisation was conducted using block design and participants were stratified according to whether they were prescribed anti-depressant medication or not. The master file was held by Stenlake pharmacy until completion of the trial. The study was registered with the Therapeutic Goods Administration, Australian Government, Trial number: 2011/0165. The study protocol was approved by the Hunter New England Human Research Ethics Committee. All mothers gave written informed consent before their participation. This informed consent included their infant.

The day before initial attendance, each participant was telephoned and completed the Edinburgh Post Natal Depression Scale (EPNDS [41]) to establish that symptoms were current, with a cutoff score of 12. On arrival a single dose of 24 IU OT or placebo nasal spray was administered. Forty-five minutes later participants completed a video-taped play session to measure Maternal Sensitivity [2]. This dose given to community women showed that saliva levels peak about 1 h after administration and are detectable 7 h later [42]. A meta-analysis of intra-nasal OT administration studies found that in the overwhelming majority of studies a dose of 24 IU was used [43]. A delay of 35–50 min between OT administration and observation of outcome was used. During the waiting time between intranasal spray and play session, on the first visit, the mothers provided written demographic and pregnancy/delivery related information. They also completed a self-report questionnaire to establish occurrence of child abuse and neglect during their past (Conflict Tactic Scales: Parent–Child Version [44]. The play sessions lasted 10 min and included 5 min each of playing with and without toys. Mothers were given the instruction to ‘play with their infant as they normally would at home’, knowing they were being videotaped. Immediately following the play session the Cry Paradigm [45] computer based rating of audio-taped newborn cries, was administered.

Participants underwent both the OT and the placebo conditions with an interval of 1 week in a balanced within-subject design. Both sessions took place within a clinical setting for families with young children (the Parent and Infant Mental Health Service, Wallsend, NSW, Australia).

### Participants

Participants with a range of social demographic factors were included in this study (see Table 1). Income levels, educational levels, age of mother and cohabitation status were all broadly represented. Three participants were using oral contraceptives (OCP).

**Table 1:** Participant characteristics

	Mean	SD	Percent
Gestational age (weeks)	38.25	3.92	
Birth weight (kg)	3.1	0.97	
Delivery: NVD^a^ (vs. Cesarian)			81.3
Gender: Female			55.6
Feeding: Breast fed (vs. bottle fed)			37.5
Family status: cohabitation (vs. single)			68.8
Annual household income: >AUD$^b^ 100,000			12.5
<AUD$ 20,000			31.3
Years of higher education	5.28	0.89	
Aboriginal			6.25
Receiving depression Rx			62.5

^a^ *NVD* normal vaginal delivery, ^b^ AUD$ Australian dollars, ^c^ *Rx* treatment

### Measures

#### Edinburgh Post Natal Depression Scale (EPNDS) [41]

This 10-item self-report screening tool to identify depression has a sensitivity of 86 % and a specificity of 78 % when used with a cutoff score of 12. A score of 12 or higher was required for inclusion into the study. Coding occurred using the directions as established by Cox et al. [41]. Scores ranged from 12 to 29 (mean 16.96, *SD* 3.41) on the first visit. Internal consistency was moderate (α = 0.62). Data inspection revealed a single outlier, which was winsorized by replacing the outlying score with a score just above the next highest value (with *z* < 3.29) [46].

#### Conflict Tactic Scales: Parent Child Version [44]

The Parent Child version of this scale was developed from a self-report instrument to measure conflict within adult relationships, the Conflict Tactic Scales. Numerous investigators have used the adaptation to establish rates of child abuse. Following the CTS guidelines, participants indicated on an 8-point rating scale the frequency of occurrence of a parental strategy in the year that they turned thirteen. An example of a prompt is “My mother threw or knocked me down”. The 22 items were coded according to the directions given in Straus [44], producing scores in the following categories: Non-violent discipline (deemed to be an optimal parenting strategy in the face of conflict), Psychological aggression, Physical aggression (separated into three levels of severity- minor corporal punishment, severe physical abuse and extreme physical abuse), and Neglect. In our sample, ‘severe physical abuse’ was spread most evenly with almost half the participants ever having experienced this form of abuse. Because of low variance for the other categories, these scales were not included in the analyses.

#### Maternal Sensitivity and Non-Intrusiveness Scales [2]

The measure consists of two 9-point rating scales (sensitivity and non-intrusiveness) describing a continuum of maternal interactive behaviors. For the sensitivity scale, a low score corresponds to low maternal sensitivity, when a mother shows extremely limited affective attunement and doesn’t seem to consider or recognize her infant as experiencing its own internal state. A high score is given to a mother who attends closely to the emotional cues given by her baby and responds in a way that maximizes the baby’s optimal level of arousal. Using the non-intrusiveness scale a low score is given to a very intrusive mother, such as frequent interference with the baby’s interests, wishes and mood. The mother behaves as though she has no respect or awareness that the baby is a separate, autonomous person. A high score is given if the mother cooperates with the activities her baby is interested in, and when needing to shift activities, this mother invites or diverts the baby, scaffolding a mood change. The video footage of the play sessions were coded independently by two trained observers, who were unaware of the condition of the mothers (OT or placebo). Intercoder reliability for sensitivity was .87, for (non-) intrusiveness it was .74 (intraclass correlation, single measure, absolute agreement, *n* = 10). We used one observer’s ratings of all first visit play sessions and the other’s ratings of all second visit play sessions.

#### Cry Paradigm

The cry paradigm was administered on a laptop using E-Prime software (Version 1.1; Psychology Software Tools, Inc., PA, USA). Previously recorded/digitally enhanced infant cry sounds were used, details of which are described by Out et al. [45]. The peak fundamental frequency of the original cry was 515 ± 15 Hz. This was subsequently digitally increased to produce two new cry sounds with overall peak frequencies of 714.5 and 895.8 Hz respectively. Higher fundamental frequencies are generally perceived as more urgent and associated with increased infant arousal [45]. The three cry sounds are referred to as the 500, 700 and 900 Hz cries. Participants listened to the presented sounds at a constant volume via Sennheiser HD 280 Pro headphones. After a trial to familiarize the participant with the equipment, cry stimuli were presented in three cycles; each cycle consisted of the three frequency stimuli. The order of presentation was random within each cycle.

Accompanying each cry sound were questions for the participant to answer, organized into two sections. Four questions pertained to the perception of the cry sound assessing the domains of arousal, aversion, sickness and urgency, as in Out et al. [45]. These domains were rated by use of a 5-point rating scale (e.g.: How urgent do you think this cry is? A score of 1 indicated “not urgent” whereas a score of 5 indicated “urgent”). As in our sample sickness showed modest item-total correlation we created a scale for Urgency based on arousal, aversion and urgency for each of the fundamental frequencies, with reliabilities reflected by Cronbach’s alphas ranging from .75 to .96 (mean .87). Nine questions pertained to the participants intended caregiving response (How likely is it that you would pick this baby up? A score of 1 indicated it “not likely”, a score of 5 indicated that it was “likely” the participant would pick up the baby in response to hearing such a cry). Caregiving response questions enquired about parenting behaviors; these were grouped into positive [to cuddle, feed, pick up the baby, focus on something else (reversed), wait and see (reversed)], and harsh (to speak firmly, shock, give the baby something to cry about) choices. We added two harsh caregiving items to the original scale used in Out et al. [45] to improve reliability and validity of the scale, as it might be of particular interest in our clinical sample. Cronbach’s alphas for intended caregiving responses to cry sounds with the three different fundamental frequencies during the two sessions separately ranged from .69 to .84 (mean .77) for positive caregiving and from .77 to .98 (mean .93) for harsh caregiving.

### Analyses

Statistical analyses were performed using SPSS 19 software. Repeated measures ANOVA were performed to evaluate the effects of oxytocin or placebo on both mothers’ sensitive behavior and cry paradigm outcomes. Experiences of physical abuse during childhood was tested as a moderator and EPNDS score tested as a covariate. In the repeated measures ANOVA, effect size (eta-squared) was computed as the ratio between sum of squares of conditions divided by the sum of squares of conditions plus sum of squares of the error component.

## Results

### Background Variables

The following background variables were not associated with mothers’ sensitive behavior or cry paradigm outcomes: baby’s age, baby’s gender, baby’s birth weight, duration of gestation, mode of delivery, and whether the mother was lactating. There were also no significant associations between maternal sensitivity or cry paradigm results and how many years of schooling the mother had received, maternal age, and whether she was taking antidepressant medication or not. Income was positively associated with observed sensitivity in the OT condition (*p* = .02), so the analyses for sensitivity were controlled for income. Similarly, the analysis on intended positive caregiving at 900 Hz was controlled for level of depression (EPNDS score at the first visit) to take the positive correlation between the two variables (*p* = .04) into account.

### Maternal Sensitivity and Intrusiveness

A multivariate repeated measures of variance was performed on the sensitivity and non-intrusiveness scales as dependent variables with condition (OT or placebo) as a within subject factor. The results were not significant (*p* = .36, *η* ^2^ = .04 for maternal sensitivity and *p* = .45, *η* ^2^ = .02 for non-intrusiveness). Income was not a significant covariate, and childhood severe physical abuse did not moderate the effect.

### Perception of Cry Sounds

A multivariate repeated measures of variance with perception as a dependent variable and condition (OT or placebo) as a within-subject factor showed a significant effect of condition for the perception of the 500 Hz cry (*F*(1,24) = 4.97, *p* = .04, *η* ^2^ = .17). Mothers rated the infant cry as more urgent in the OT condition as compared to the placebo condition. Experiences of physical abuse during childhood did not moderate the effect. OT did not significantly affect the perception of the 700 Hz cry or the 900 Hz sound and experiences of physical abuse during childhood did not moderate results for these frequencies either.

### Intended Care Giving

**Fig. 1:**
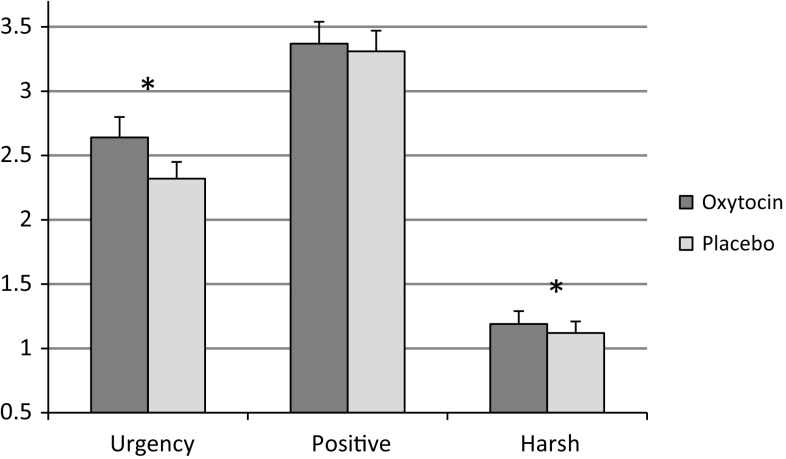
The effect of oxytocin on cry perception and intended caregiving at 500 Hz (mean, SE). Urgency—perceived urgency of cry, Positive—intended positive caregiving, Harsh—intended harsh caregiving. *p < .05

For intended positive caregiving, we did not find an effect of OT for the 500 Hz cry sound, *F*(1,24) = 0.32, *p* = .58, *η* ^2^ = .01. For harsh caregiving however, mothers were more likely to be harsh in the OT condition, *F*(1,24) = 5.60, *p* = .03, *η* ^2^ = .19 (see Fig. 1). Nineteen percent of the variance in intended harsh caregiving is explained by the OT condition. Results were not moderated by a history of childhood physical abuse. For the other fundamental frequencies (700 and 900 Hz) no effects of OT administration were found. Again findings were not moderated by childhood physical abuse and EPNDS score was not a significant covariate.

## Discussion

The majority of our findings were not significant. However the combination of our significant findings, in this vulnerable population, are concerning. We found that in the OT condition, mothers with a diagnosis of PND rated a naturalistic newborn cry as more urgent than in the placebo condition. We also found that in the OT condition subjects in this clinical sample were more likely to choose a harsh caregiving strategy in response to the 500 Hz cry sound. There was no effect of OT on observed maternal sensitivity.

The combination of perceiving a cry as more urgent but choosing a harsh strategy might indicate that OT enhances a depressed mother’s already negative cognitive processes, as has been found previously. In studies on non-clinical subjects, OT seemed to increase the salience of emotional states [36, 47–49]. In this clinical sample, mothers were more likely to describe their babies as more difficult and reported a lower mood in the OT condition [39] compared to the placebo condition. Of our findings, the most salient was the significant increase in a harsh caregiving choice when asked how the mother imagined she would respond to a cry. The effect size was substantial, 19 % of the variance was due to the OT condition. Of course it is important to consider if this finding is clinically significant, given the relatively small magnitude of increase in intended harsh care-giving and the statistical uncertainty of the point estimate in this small sample. Magnitude to determine clinical significance is debatable, but given the child protection relevance of potential harm to an infant, this finding is important to consider carefully. Other published studies may help us to understand the direction of this finding. OT has been found to increase ‘non-cooperation’ when a partner is considered a threat in studies using games to assess trust and cooperation in community adults [38]. It may be that a depressed mother is more likely to view a crying infant as some form of threat, even if just to her emotional well-being.

Also worthy of consideration is the fact that in our study there were only significant findings for one of the fundamental frequencies tested, 500 Hz. The influence of genetic factors and cry pitch was investigated using the cry paradigm in a community sample [45] with the same cry sounds. Significant findings showed that adults were more likely to choose a sensitive intended caregiving strategy for the higher pitched cries and when they perceived the cry as more urgent. The 500 Hz cry was a naturalistic recording and both the 700 Hz and the 900 Hz cry were digitally altered to achieve higher pitched cry sounds with identical temporal structure in order to study the effects of cry pitch on adults’ perception and caregiving responses. Future research should also use naturalistic high pitched cry recordings to answer the question whether OT has effects in depressed mothers when responding to naturalistic high pitched cry sounds or only at lower pitches.

OT administration did not impact on maternal sensitivity in our clinically depressed sample. What is interesting to consider is the different findings from our study compared to a very similar study using a community sample of fathers [35]. In Naber et al. significant improvements in parent child interactions were found in a smaller sample of seventeen fathers in a within subject, randomized placebo controlled design using an identical OT dose and duration of administration to outcome measure. Based on the effect size reported in the Naber et al. study, the power to find a significant effect in our sample was 78 % for an expected positive effect of OT administration on parenting sensitivity. The absence of a significant effect on sensitivity in our sample adds some weight to the current trend found in two recent meta-analytic studies that OT effects are often diminished or absent in clinical samples [38, 49]. One mechanism proposed by Bakermans-Kranenburg et al. [38] was that early environmental factors which may predispose an individual to depression may cause changes to the OT system by methylation of associated genes. Future research could explore this idea further.

Contrary to our hypotheses, the presence of severe physical abuse during the childhoods’ of our participants did not moderate the OT effect in the outcome measures studied. Previous studies finding differential susceptibility to the effects of OT have used various measures of childhood adversity [37, 38, 40]. Bakermans-Kranenburg et al. [37] used the same measure as in the current study, but an alternate subscale, that of harsh discipline. In our sample the most evenly represented domain was severe physical abuse. We were unable to determine if harsh discipline was a potential moderating factor due to the low variance of scores on this construct in our sample. Thus, a more direct comparison using the same subscale as Bakermans-Kranenburg et al. could not be undertaken. Other limitations of our study should also be considered. Our outcome measure of self-reported intended caregiving response could be improved by using observational methods in future studies. Under-reporting of intended harsh caregiving strategies may have occurred due to social desirability [50].

Strengths of our study include its sound research design. Using a within-subject design increases the power without having to expose a larger number of clinically depressed participants to a hormonal manipulation, an ethically important consideration –although negative side-effects have not been reported so far [51].

## Summary

In conclusion, this study found that OT has significant effects on the perception of urgency and on the choice of a parenting strategy in response to infant cry sounds in a sample of mothers with a diagnosis of PND. We found no effect of OT on maternal sensitivity. The finding of increased intended harsh caregiving is important and we should fully explore this association prior to using OT as a pharmacotherapy to enhance parental capacity, especially in a depressed population, as we may induce an iatrogenic effect.

## Abbreviations

EPNDS: Edinburgh postnatal depression scale
OCP: Oral contraceptive pill
OT: Oxytocin
PND: Postnatal depression
